# Reproducibility for Hepatocellular Carcinoma CT Radiomic Features: Influence of Delineation Variability Based on 3D-CT, 4D-CT and Multiple-Parameter MR Images

**DOI:** 10.3389/fonc.2022.881931

**Published:** 2022-04-14

**Authors:** Jinghao Duan, Qingtao Qiu, Jian Zhu, Dongping Shang, Xue Dou, Tao Sun, Yong Yin, Xiangjuan Meng

**Affiliations:** ^1^ School of Precision Instrument and Opto-electronics Engineering, Tianjin University, Tianjin, China; ^2^ Department of Radiotherapy, Shandong Cancer Hospital and Institute, Shandong First Medical University and Shandong Academy of Medical Sciences, Jinan, China; ^3^ Department of Clinical Laboratory, Shandong First Medical University and Shandong Academy of Medical Sciences, Shandong Cancer Hospital and Institute, Jinan, China

**Keywords:** radiomic feature, reproducibility, hepatocellular carcinoma (HCC), multiple-parameter MR images, 4D-CT

## Abstract

**Purpose:**

Accurate lesion segmentation is a prerequisite for radiomic feature extraction. It helps to reduce the features variability so as to improve the reporting quality of radiomics study. In this research, we aimed to conduct a radiomic feature reproducibility test of inter-/intra-observer delineation variability in hepatocellular carcinoma using 3D-CT images, 4D-CT images and multiple-parameter MR images.

**Materials and Methods:**

For this retrospective study, 19 HCC patients undergoing 3D-CT, 4D-CT and multiple-parameter MR scans were included in this study. The gross tumor volume (GTV) was independently delineated twice by two observers based on contrast-enhanced computed tomography (CECT), maximum intensity projection (MIP), LAVA-Flex, T2W FRFSE and DWI-EPI images. We also delineated the peritumoral region, which was defined as 0 to 5 mm radius surrounding the GTV. 107 radiomic features were automatically extracted from CECT images using 3D-Slicer software. Quartile coefficient of dispersion (QCD) and intraclass correlation coefficient (ICC) were applied to assess the variability of each radiomic feature. QCD<10% and ICC≥0.75 were considered small variations and excellent reliability. Finally, the principal component analysis (PCA) was used to test the feasibility of dimensionality reduction.

**Results:**

For tumor tissues, the numbers of radiomic features with QCD<10% indicated no obvious inter-/intra-observer differences or discrepancies in 3D-CT, 4D-CT and multiple-parameter MR delineation. However, the number of radiomic features (mean 89) with ICC≥0.75 was the highest in the multiple-parameter MR group, followed by the 3DCT group (mean 77) and the MIP group (mean 73). The peritumor tissues also showed similar results. A total of 15 and 7 radiomic features presented excellent reproducibility and small variation in tumor and peritumoral tissues, respectively. Two robust features showed excellent reproducibility and small variation in tumor and peritumoral tissues. In addition, the values of the two features both represented statistically significant differences among tumor and peritumoral tissues (*P*<0.05). The PCA results indicated that the first seven principal components could preserve at least 90% of the variance of the original set of features.

**Conclusion:**

Delineation on multiple-parameter MR images could help to improve the reproducibility of the HCC CT radiomic features and weaken the inter-/intra-observer influence.

## Introduction

Liver cancer has been the third leading cause of cancer-related death worldwide, totaling nearly 905,700 new cases in 2020 (1). In some areas, the mortality rate is almost equal to the morbidity rate (2). Hepatocellular carcinoma (HCC) is the most common histologic subtype of liver cancer, accounting for more than 90% of all cases. Liver cancer treatment has been evolved from surgery, interventional therapy, radiotherapy, chemotherapy, targeted therapy to immunotherapy (3). However, early detection is almost the only chance of long-term survival for HCC patients. In addition, accurate prediction and evaluation of the profile of patients are also an important pathway to improve their survival rate.

Noninvasive imaging (i.e., ultrasound, computed tomography (CT), magnetic resonance imaging (MRI), etc.) plays an important role in the characterization and monitoring of liver cancer. The modern (or advanced) imaging technology has allowed qualitative evaluation of liver cancer (or other liver diseases) and assisted personalized medical decision-making (4, 5).

Thereinto, radiomics is an innovative technology deriving from early 2012 (6), serving as the preferred method to study the relationship between cancer imaging phenotypes, cancer genotypes and clinical prognosis (7–9). It refers to the high-throughput extraction of numerous quantitative and mineable imaging features assumed to convey prognostic and predictive information. The most prominent advantage is that it realizes objective non-invasive assessment of tumors using conventional imaging data, including heterogeneity, as opposed to current visual identification.

Although strong evidence shows that radiomics contributes to clinical decisions, no clinical application of radiomic features has been developed to date. Some scholars suggest that radiomics studies must be cautiously approached because several radiomic features change significantly with slight variation in images (10, 11). Therefore, in each step of the radiomics workflow, many challenges exist for clinical transition, including but not limited to the interpretability of models, reproducibility of radiomic features, and sensitivity to changes in image acquisition, reconstruction parameters, and tumor segmentation (12). A main challenge is the lack of reproducibility regarding the reported radiomic features and models (13). The search engine PubMed showed that investigations of feature reproducibility or repeatability were only limited to a small number of cancer types (14). Reproducibility can be pursued in imaging data, segmentation, calculations or statistics, and studies (11, 15), among which segmentation is considered the most critical, challenging and controversial part of radiomics analysis (11).

For the past few years, several studies involving lung tumor, head and neck tumor, renal tumor, liver tumor, and pancreas tumor have reported the impact of target segmentation on the reproducibility of radiomic features (16–19). These investigations suggest that the minimization of the observer delineation is an effective way for improving the reproducibility of radiomics features, which can be well achieved by supervising computed results, performing inter/intra-reader variability, obtaining the consensus of radiologists on contouring and using the software properly (20). Another notable approach is multimodal imaging. Although it can provide a better region-of-interest (ROIs) delineation, evidence is scarce as to whether it can increase the reproducibility of the radiomics features.

Accurate lesion segmentation is a prerequisite for radiomic feature extraction. However, the liver lesion delineation represents a great variation on CT images, even in contrast-enhanced computed tomography (CECT) images. The magnetic resonance (MR) technique can significantly increase the discrimination of liver lesion boundaries.

In view of this, we aimed to carry out a reproducibility test of radiomics features of inter-/intra-observer delineation variability in HCC using CT images, 4DCT images and multiple-parameter MR images. This study is expected to provide a new approach to improve the reproducibility of radiomics features.

## Materials and Methods

### Patient Population

This study was approved by the local ethics committee. Considering the present observational and retrospective study using a database, informed consent was waived by our ethics institution. The identifying information of each patient had been removed. A database of 211 HCC patients treated with radiotherapy was identified between September 2018 and April 2021 in Shandong Cancer Hospital and Institute. Imaging follow-up was obtained through the Eclipse treatment planning system (TPS) (Varian Medical Systems, Palo Alto, CA). Eligibility criteria were as follows: (I) Cases were diagnosed with HCC by pathology; (II) the tumor diameter was greater than 5 mm, and at least two slices showed visible lesions; (III) suggested by ESUR guidelines on contrast agents (21), glomerular filtration rate (GFR) was more than 30 mL/min/1.73 m^2^. Exclusion criteria were as follows: (I) Liver metastasis; (II) poor image quality; (III) data partial missing; (IV) surgical resection; (V) history of radiation therapy; (VI) liver transplant; (VII) poor image registration results between CT and MR images.

### Image Acquisition

All patients underwent the liver CT scan (Brilliance iCT 128, Phillips Medical Systems, Netherlands) and 3.0T MRI scan (Discovery 750 W, General Electric Co., Boston, USA). The CECT was performed before enhanced MR. During CT scans, patients were placed supine with their arms above their heads and immobilized using an evacuated cushion. All patients sequentially acquired 3D and 4D CT scans while breathing freely. The 3D-CT signal intensity was collected in arterial phase images. Based on the respiratory signal obtained from the real-time location management system (Varian Medical Systems, Palo Alto, USA), 10 respiratory phases (i.e., CT00, CT10, CT20, CT30, CT40, CT50, CT60, CT70, CT80, CT90) were acquired, among which CT00 and CT50 were defined ned as end-inhalation and end-exhalation, respectively. The maximum intensity projection (MIP) images were immediately acquired. The matrix size was 512×512 with a pixel spacing of 0.97×0.97×3.0 mm^3^ in the left-right, antero-posterior and craniocaudal directions, respectively.

To reduce any potential for nephrotoxicity, MR scans were subsequently conducted 4 hours after CT scans on the same day. During MR scans, patients were immobilized on the patient table overlaid with a flat couch top with the identical dedicated device and in the same position as in CT scans. Before scanning, all patients underwent a bolus injection of gadoteric acid (0.2 mmol/kg, 2 mL/sec) and immediately a 20 mL saline flush. Three pulse sequences of MR simulation images were acquired: (1) Axial LAVA-Flex: repetition time (TR)=5 ms, echo time (TE)=2 ms, slice thickness=3 mm, interslice spacing=0 mm, NEX=0.71, FOV=44 cm×35.2 cm, matrix size=296×256; (2) axial T2W FRFSE: repetition time (TR)=11250 ms, echo time (TE)=87.9 ms, slice thickness=3 mm, interslice spacing=0 mm, NEX=2.5, FOV=44 cm×44 cm, matrix size=384×384; (3) DWI-EPI: repetition time (TR)=18750 ms, echo time (TE)=60.3 ms, slice thickness=4 mm, interslice spacing=0 mm, NEX=1, FOV=44 cm×35.2 cm, matrix size=96×128; b=800 s/mm^2^.

### ROIs Delineation

Based on the images of CECT, MIP, DWI and T2W FRFSE, the gross tumor volume (GTV) was independently delineated twice (ten days apart) by two observers (an experienced radiation oncologist and a radiologist). The MIP delineation results, as well as the merged DWI and T2W FRFSE delineation results by rigid registration, were copied on CECT images. Automatic grayscale registration was performed and manually corrected according to the outer contours of the liver. The structures delineated by the first and second observers were named 3DCT1, MIP1, MR1, 3DCT2, MIP2, MR2, 3DCT3, MIP3, MR3, 3DCT4, MIP4, and MR4 for the first and second delineations, respectively.

In addition, we also delineated the peritumoral region, which was defined as a 0 to 5 mm radius surrounding the GTV.

### Feature Extraction

All features were extracted from CECT images by using PyRadiomics platform implanted in the 3D Slicer software. (v4.8.1), a popular open-source platform for the processing and analysis of medical images. Radiomics features were extracted from original image. All textural features were discretized using absolute gray level discretization with a fixed bin width value of 25. A total of 107 radiomic features were extracted and divided into 6 groups according to the feature calculation method: first-order, gray level co-occurrence matrix (GLCM), gray level dependence matrix (GLDM), gray level run length matrix (GLRLM), gray level size matrix (GLSZM), neighborhood gray-tone difference matrix (NGTDM) and shape. A total of 18, 24, 14, 16, 16, 5, and 14 features were extracted for each matrix, respectively. The definitions and interpretations of these features have been reported on this website (https://pyradiomics.readthedocs.io/en/latest/features.html).

### Data Analysis and Statistics

#### Variation of Radiomics Features

Quartile coefficient of dispersion (QCD) was used to assess the variation of radiomics features. QCD<10% was considered small variations, 10%≤QCD<20% was considered intermediate variations, and QCD≥20% was considered large variations. The definition of QCD was as the equation 1:


(1) $$QCD=\frac{Q3-Q1}{Q3+Q1}\times 100$$

where Q1 and Q3 denote the first and third quartiles (22), respectively.

#### Reproducibility of Radiomics Features

The inter-/intra-observer reproducibility of radiomics features was evaluated using the intraclass correlation coefficient (ICC), a primary metric in most previous research (16–19). The ICC was calculated as the equation 2:


(2) $$ICC=\frac{MS_{R}-MS_{E}}{MS_{R}+(k-1)MS_{E}+\frac{k}{n}(MS_{C}-MS_{E})}$$

where *MS_R_* means the mean square for rows (observations), *MS_E_* denotes the mean square error, *MS_C_* represents the mean square for columns and *k* is the number of raters (normalization methods or observers). ICC=0 indicates no reliability, while ICC=1 represents highly stable features. We adopted the previous reports to interpret the ICC interrater agreement measures (23): ICC values less than 0.4 indicate poor reliability, values between 0.4 and 0.59 indicate fair reliability, values between 0.6 and 0.74 indicate good reliability, and values greater than 0.75 indicate excellent reliability.

### Identification of Tumor Tissues and Peritumor Tissues Using Robust Radiomic Features

The radiomic features with ICC>0.75 and QCD<10% in both tumor and peritumoral tissues were selected to identify the differences between the two groups. The two groups were compared using one-way ANOVA or Kruskal-Wallis test. *P*<0.05 was considered statistically significant. The SPSS 20.0 software and GraphPad Prism 9.2 were used to do all statistical analyses.

### Radiomic Features Dimensionality Reduction

In addition, for CT radiomic features with ICC≥0.75 based on the ROIs delineation of multiple-parameter MR images, principal component analysis (PCA) was performed to test the feasibility of dimensionality reduction. PCA is widely used for qualitative analysis and data reduction to explore data characteristics. To preserve at least 90% of variances of the original feature set, the number of principal components was detected.

## Results

### Study Cohort

**Figure 1:**
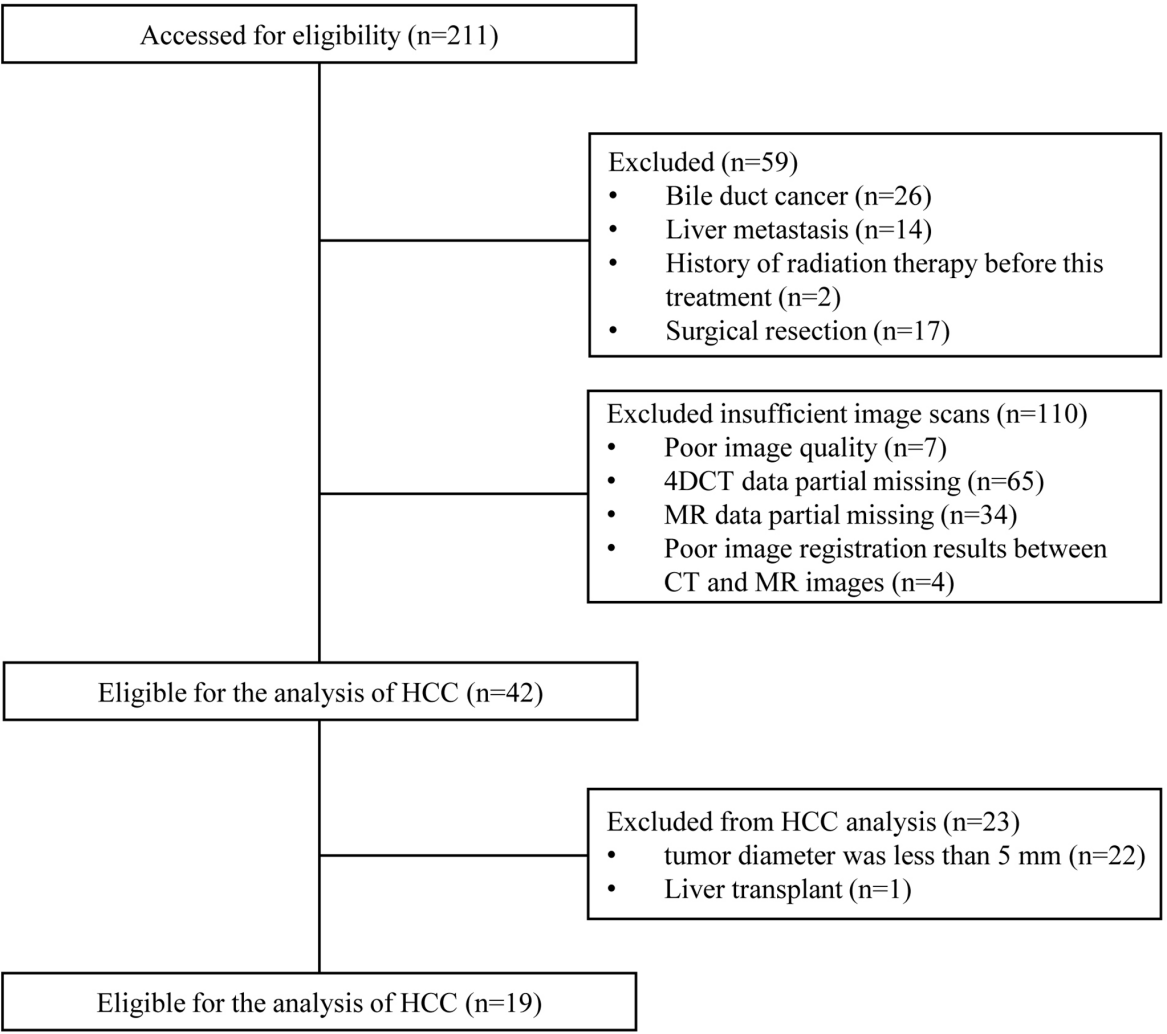
Flow diagram for case selection.

**Figure 2:**
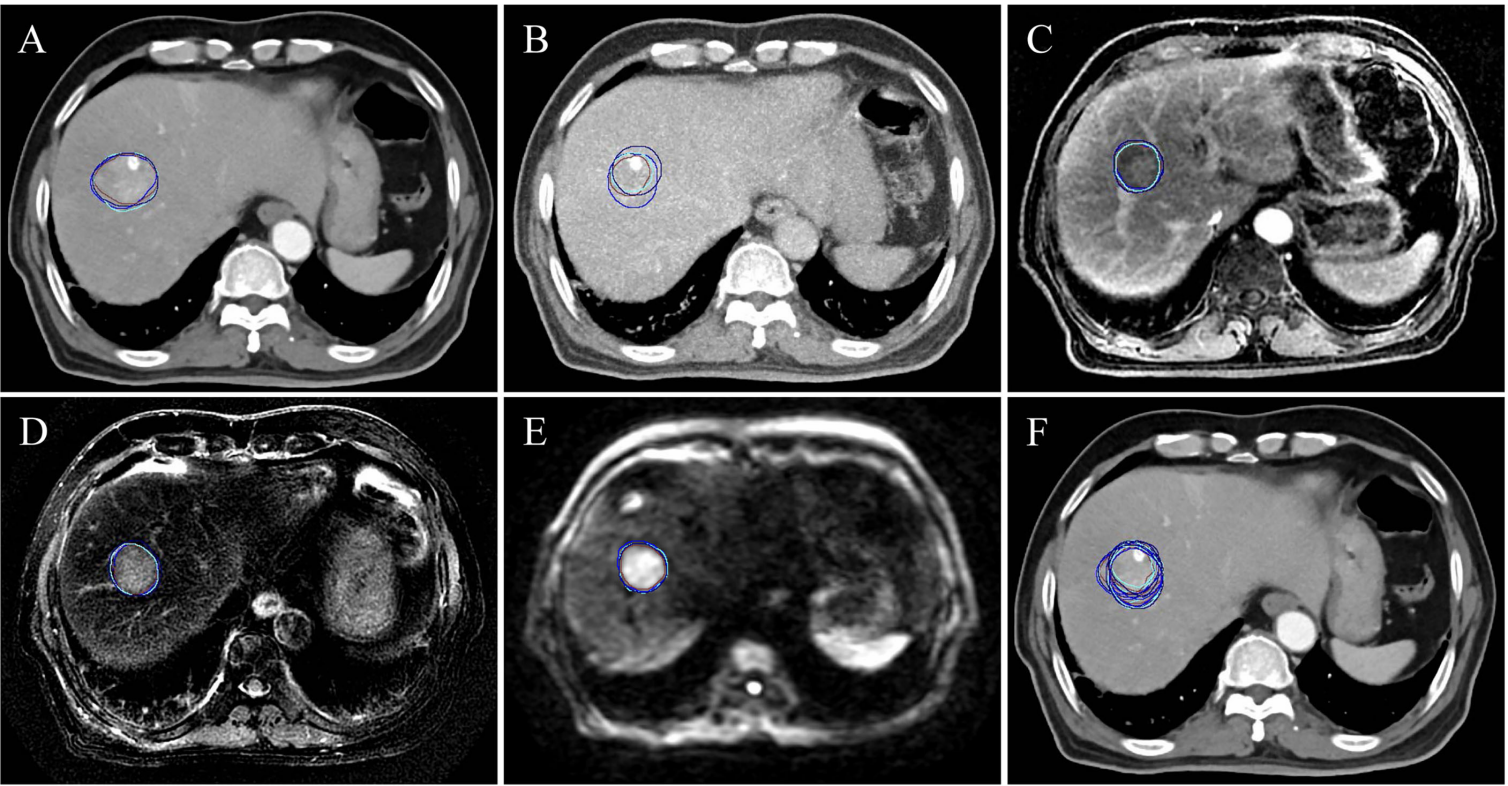
A subject (a male aged 69 and diagnosed with hepatocellular carcinoma) of the target delineation for primary liver cancer in 3D-CT, MIP and multiple-parameter MR images. **(A-E)** show the delineation results of CECT, MIP, LAVA-Flex, T2W FRFSE and DWI-EPI, respectively. **(F)** shows all the delineation results displayed on CECT. The blue, brown, cyan and dark blue contour denote the first delineation of the first observer. The brown contour is the second delineation of the first observer. The cyan contour is the first delineation of the second observer. The dark blue is the second delineation of the second observer.

A flowchart of patient participation and sample selection is given in **Figure 1**. Finally, 19 HCC patients (15 males and 4 females aged from 45 to 75 with mean age of 57.4 years) were included in this study. The smallest, mean and largest tumor volume is 19.5 cm^3^, 105.3 cm^3^ and 385.4 cm^3^ respectively. A total of 456 contouring results were obtained for this research (228 contouring results for tumor and peritumoral region, respectively). **Figure 2** illustrates an example of the target delineation for HCC in 3D-CT, MIP and MR images.

### Variation of Radiomic Features According to QCD

For tumor tissues, the numbers of radiomic features with QCD ≤ 10% in 3DCT1, 3DCT2, 3DCT3, 3DCT4, MIP1, MIP2, MIP3, MIP4, MR1, MR2, MR3 and MR4 were 15, 13, 10, 15, 14, 13, 11, 13, 15, 12, 12 and 12, respectively. For peritumoral tissues, the numbers of radiomic features with QCD ≤ 10% in 3DCT1, 3DCT2, 3DCT3, 3DCT4, MIP1, MIP2, MIP3, MIP4, MR1, MR2, MR3 and MR4 were 14, 11, 12, 14, 13, 12, 12, 11, 13, 16, 13 and 13, respectively. A detailed QCD results can be found in **Supplementary Materials** (see **Table S1**).

### Reproducibility of Radiomics Features According to ICC

**Figure 3:**
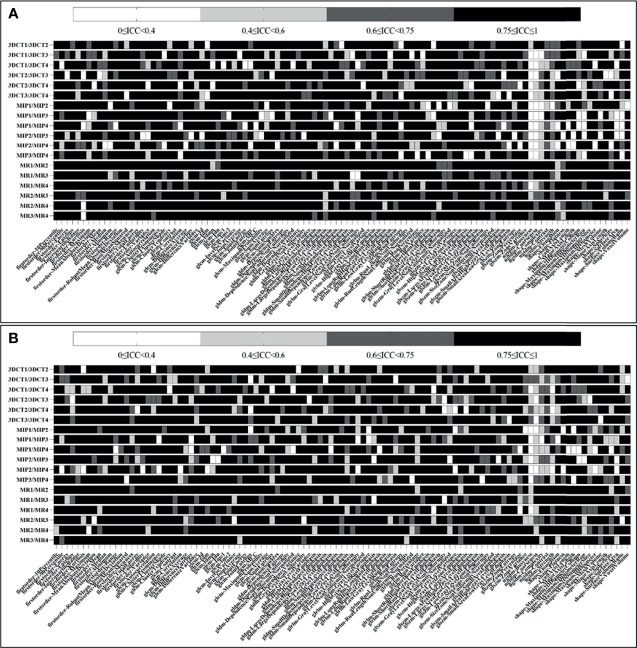
Heatmap demonstrating tumor and peritumor tissue-derived reproducible features, where each row and column represent each comparison dataset and radiomic feature, respectively. **(A, B)** show the ICC results of tumor and peritumor tissues, respectively.

As seen in **Figure 3A**, for tumor tissue-derived radiomic features, when ICC≥0.75 is the threshold, 85/107 (79.4%), 72/107 (67.3%), 70/107 (65.4%), 74/107 (69.2%), 77/107 (72.0%), 82/107 (76.6%), 77/107 (72.0%), 75/107 (70.1%), 70/107 (65.4%), 71/107 (66.4%), 69/107 (64.5%), 77/107 (72.0%), 99/107 (92.5%), 84/107 (78.5%), 82/107 (76.6%), 85/107 (79.4%), 90/107 (84.1%), and 96/107 (89.7%) features meet the threshold for 3DCT1/3DCT2, 3DCT1/3DCT3, 3DCT1/3DCT4, 3DCT2/3DCT3, 3DCT2/3DCT4, 3DCT3/3DCT4, MIP1/MIP2, MIP1/MIP3, MIP1/MIP4, MIP2/MIP3, MIP2/MIP4, MIP3/MIP4, MR1/MR2, MR1/MR3, MR1/MR4, MR2/MR3, MR2/MR4, and MR3/MR4, respectively. The mean numbers of radiomic features with ICC≥0.75 in intra-observer groups are much more than those in inter-observer groups using CECT (84/107 (78.5%) *vs*. 74/107 (69.2%)), MIP (77/107 (72.0%) *vs*. 71/107 (66.4%)) or MR (98/107 (91.6%) *vs*. 85/107 (79.4%)) delineations. Notably, the excellent reproducibility numbers in MR delineations are much more than those in CECT delineations in both intra-observer groups (98/107(91.6%) *vs*. 84/107(78.5%)) and inter-observer groups (85/107(79.4%) *vs*. 74/107(69.2%)). The excellent reproducibility numbers in MIP delineations are the least in both groups. **Supplementary Material** (**Figure S1**) shows the ICC values in each radiomic features subgroup for CECT, MIP and MR delineations.

As seen in **Figure 3B**, for peritumoral tissue-derived radiomic features, the results are similar to those of tumor tissues. The information of the radiomic features subgroup is shown in the **Supplementary Material** (**Figure S2**).

### Radiomic Features With Small Variation and Excellent Reproducibility

**Figure 4:**
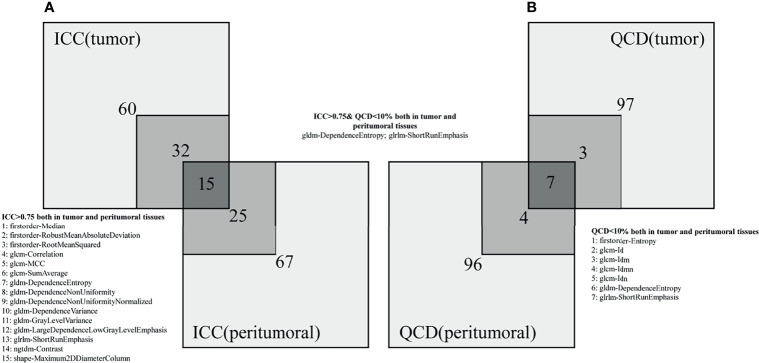
Numbers of radiomic features not affected by intra-/inter-observer MR delineations. **(A, B)** represent the ICC and QCD, respectively.

**Figure 4** indicates that radiomic features are not affected by the intra-/inter-observer MR delineations. The 47/107 (43.9%) and 40/107 (37.4%) features show excellent reproducibility for tumor and peritumoral tissues, respectively. Fifteen radiomic features present excellent reproducibility in both tissues. Ten and eleven radiomic features show small variations for tumor and peritumoral tissues, respectively. Seven radiomic features present small variations in both tissues. Only 2 robust features (GLDM-dependence entropy and GLRLM short run emphasis) show excellent reproducibility and small variation in both tissues. Names of specific radiomic features can be found in **Supplementary Materials** (see **Table S2**).

### Identification of Tumor and Peritumor Tissues

**Figure 5:**
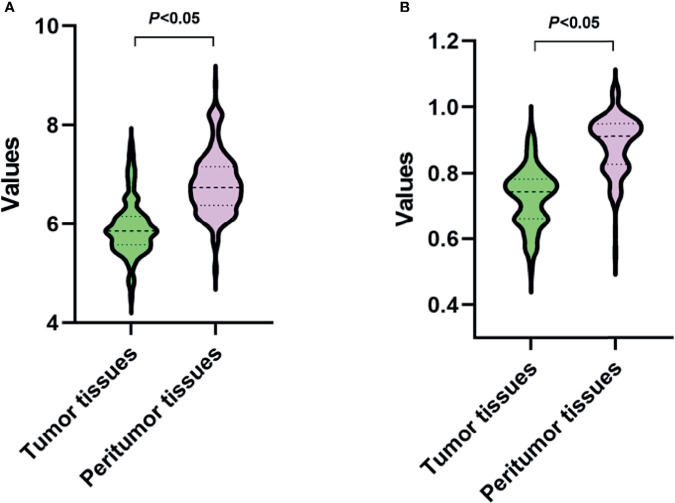
Values of two robust radiomic features in tumor and peritumor tissues. **(A, B)** present the features of GLDM-dependence entropy and GLRLM short run emphasis, respectively.

The robust features were used to identify the tumor and peritumor tissues. As shown in **Figure 5**, in the GLDM-dependence entropy feature, the mean values of tumor and peritumor tissues are 5.93 and 6.81, respectively. In the GLRLM short run emphasis feature, the mean values of tumor and peritumor tissues are 0.73 and 0.89, respectively. Taken together, both features showed statistically significant differences among the two groups (*P*<0.05).

### Radiomic Features Dimensionality Reduction

**Figure 6:**
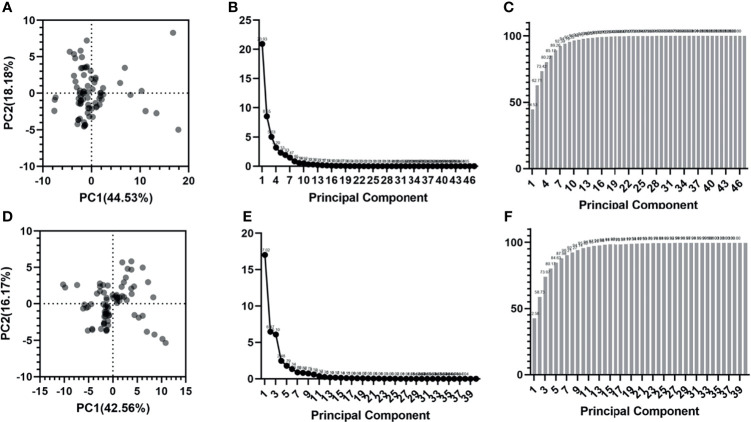
Radiomic features dimensionality reduction using PCA. **(A-C)** are the score plot, scree plot and proportion of variance plot for tumor tissue-derived radiomic features. **(D-F)** are the score plot, scree plot and proportion of variance plot for peritumoral tissue-derived radiomic features.

The PCA results can be seen in **Figure 6**. For tumor tissue-derived radiomic features, the variance contribution rates of the first principal component (PC1) and the second principal component (PC2) are 44.53% and 18.18%, respectively. The eigenvalues for each principal component are shown in **Figure 6B**. The cumulative proportion of variance is presented in **Figure 6C**. The first seven principal components can preserve at least 90% (92.39%) of the variance of the original feature set.

For peritumoral tissue-derived radiomic features, the variance contribution rates of PC1 and PC2 are 42.56% and 16.17%, respectively. The eigenvalues for each principal component are listed in **Figure 6E**. The cumulative proportion of variance is shown in **Figure 6F**. The first seven principal components can preserve at least 90% (90.21%) of the variance of the original feature set.

## Discussion

The term “radiomics” has been coined for almost a decade since 2012. Despite numerous published studies, few are put into clinical practice because of the gaps in radiomics research (24). The gaps are multifaceted, sourced from the following parts (13): (1) features selection; (2) model building; (3) model selection; (4) model validation; (5) model performance; (6) intended clinical use; (7) impact on outcomes. In 2017, a consensus statement (25) and review (26) pointed the way to accelerate the clinical translation of radiomics, in which optimal reproducibility and stability of radiomic features is one necessary component but far from being fit-for-purpose in routine clinical use.

Recently, the sources of variation radiomics features were comprehensively surveyed (20). The results revealed that multiple sources of variation exist in each step of the radiomics workflow, starting with the earliest radiomics analyses. For example, features may be sensitive to heterogeneous image acquisition settings (scanners, scanning techniques, image filtering, and reconstruction parameters) (10, 27, 28), organ motion (29), tumor types and uncertain tumor boundaries in the segmentation step (inter-/intra-algorithm and post-seg. correction). Moreover, potential strategies and practical considerations were also discussed for reducing feature variability and improving the quality of radiomics studies. The inter-reader (or intra-reader) testing was suggested to identify radiomic features sensitive to lesion segmentation so that these features could be removed from subsequent analyses.

As described in the introduction section, several studies have reported the impact of tumor segmentation variants on the reproducibility of the radiomic features. Matea et al. (16) investigated the impact of inter-observer variability in manual tumor delineation on the reliability of radiomic features. They found that differences in tumor delineation could exert relevant influences on the results of a radiomics analysis. Furthermore, this effect varied with types of tumors. Brook et al. (17) identified that radiomic features were sensitive to even a slightly 2-mm change in segmentation margin for clear cell renal cell carcinomas. The possible influence of the segmentation margin was suggested for consideration. In addition, the reproducibility of the radiomic features significantly relied on the tumor segmentation (manually, semi-automatically, and fully-automated) and scan parameters (18, 19). In MR images, Alberto et al. (30) evaluated the stability of radiomic features obtained from apparent diffusion coefficient (ADC) maps of cervical cancer. They found that shape features were potentially more prone to inter-observer variability, and normalization prior to feature extraction contributed to increasing the reproducibility of ADC-based radiomics features. To sum up, all the above studies were performed in a single modality image during tumor segmentation, ignoring the value of multimodal images. In fact, in tumor delineation, multimodal images (such as PET-CT, multiple-parameter MR images, and CECT) can reduce the delineation variation, especially in HCC (31–33). However, the reproducibility of the resulting radiomic features remains unclear so far.

This study investigated the reproducibility of CT radiomic features from HCC *via* multimodal images target delineation. We found the numbers of excellent reproducibility for multiple-parameter MR images delineation were much more than those of CTCT and MIP images delineation in inter- and intra-observer contouring. The delineation of MIP images supported the least excellent reproducibility features. The reason might be that MR images could provide a high-contrast tumor than CECT images. The tumor borders were difficult to be defined in MIP images.

To the best of our knowledge, this study blazed a trail in assessing the radiomic feature variation stemming from different modal images. In addition, the differences between intra- and inter-observer results were also analyzed. More importantly, our research proved that this method could effectively improve the reproducibility of CT radiomics features in liver cancer.

Many tumors have indistinct borders, posing challenges to the reproducibility of their delineation. MRI has a significantly higher agreement on ROI delineation than CT images in some tumors, such as brain tumor (34), prostate cancer (35), and HCC (36). Therefore, this study tried to use multiple-parameter MR images to delineate the liver tumor. The radiomic feature extraction was performed on CT images. MR images have an obvious limitation in studying the reproducibility of radiomic features because of numerous scanning parameters. The magnetic field strength, b-values, radiofrequency coil, corrections for gradient non-linearity and other factors presented large variations. Sandra et al. (37) reported that few reproducible MRI-based radiomic features were acquired in cervical cancer, and shape features showed the most reliability. Another study (38) showed few reproducible radiomic features extracted from T1W and T2W imaging. In addition, normalization could also impact the reproducibility of T2W-MRI radiomic features (39). However, FLAIR imaging and T2 mapping could provide a large number of robust radiomic features (38, 40). CT images had fewer parameter settings, and radiomic features were hardly dependent on the time after contrast injection (41). Therefore, to date, most studies focused on radiomics analyses of CT, which is thus utilized in this study to perform radiomic features extraction.

Responsible radiomics research is crucial for advancing clinical translation, which requires great efforts to improve radiomics reproducibility. Choe et al. attempted to improve the reproducibility of radiomic features by applying kernel transformation techniques based on the convolutional neural network (42). This study takes the first step toward bigger strides in radiomics. Therefore, we have reason to believe that deep learning in the future will reverse the current research on the reproducibility of radiomics. Furthermore, to increase consistency in tumor delineation, automatic and semi-automatic precision segmentation algorithms are urgently needed (43, 44). Of course, the universality of algorithms needs to be confirmed by multiple observers in different fields. Insufficient transparency in reporting radiomics studies hinders the translation. Data sharing and open-source software packages are indispensable in the future to prove the clinical usefulness of radiomics (45, 46).

Our study also had several limitations. Firstly, this study was conducted at a single hospital, and the distribution of liver pathological changes and ethnic trends might differ slightly from other institutions. The multi-center study will be embraced in future work. Secondly, HCC presents high heterogeneity. Several studies have shown that radiomics can help unravel information about tumor heterogeneity hiding in medical images. If the radiomic features were extracted from the whole tumor, the reproducibility might be influenced by the HCC heterogeneity to some extent. Another limitation was that differences in tumor contours due to multimodal image registration algorithms were not considered (such as deformation registration versus rigid registration), which should be the focus of future studies.

## Conclusion

We provide a new strategy to screen more reproducible radiomic features in HCC. Delineation on multiparametric MRI images in liver tumors and the subsequent extraction on CT images can help to capture more reproducible radiomic features.

## Data Availability Statement

The original contributions presented in the study are included in the article/**Supplementary Material**. Further inquiries can be directed to the corresponding author.

## Ethics Statement

Written informed consent was obtained from the individual(s) for the publication of any potentially identifiable images or data included in this article.

## Author Contributions

Conceived and designed the experiments: JD and XM. Performed the experiments: JD, QQ. Analyzed the data: all authors. Wrote the paper: JD and XM. All authors contributed to the article and approved the submitted version.

## Funding

This research was partly supported by the National Nature Science Foundation of China (No. 81901743, No. 82001902 and No. 82172072), the Natural Science Foundation of Shandong Province (No. ZR2020QH198 and ZR2020LZL001).

## Conflict of Interest

The authors declare that the research was conducted in the absence of any commercial or financial relationships that could be construed as a potential conflict of interest.

## Publisher’s Note

All claims expressed in this article are solely those of the authors and do not necessarily represent those of their affiliated organizations, or those of the publisher, the editors and the reviewers. Any product that may be evaluated in this article, or claim that may be made by its manufacturer, is not guaranteed or endorsed by the publisher.

## Supplementary Material

Supplementary Figure 1 The ICC values in each radiomic feature subgroup for CECT, MIP and MR delineations for tumor tissues.

Supplementary Figure 2 The ICC values in each radiomic feature subgroup for CECT, MIP and MR delineations for peritumoral tissues.

Supplementary Table 1 The detailed QCD numbers of radiomic features.

Supplementary Table 2 The specific radiomic feature with small variation and excellent reproducibility.
