# Supplementary figures and images for: Reproducibility for Hepatocellular Carcinoma CT Radiomic Features: Influence of Delineation Variability Based on 3D-CT, 4D-CT and Multiple-Parameter MR Images
Source: Front Oncol. 2022 Apr 14;12:881931. doi: 10.3389/fonc.2022.881931 (PMC9047864; doi:10.3389/fonc.2022.881931)

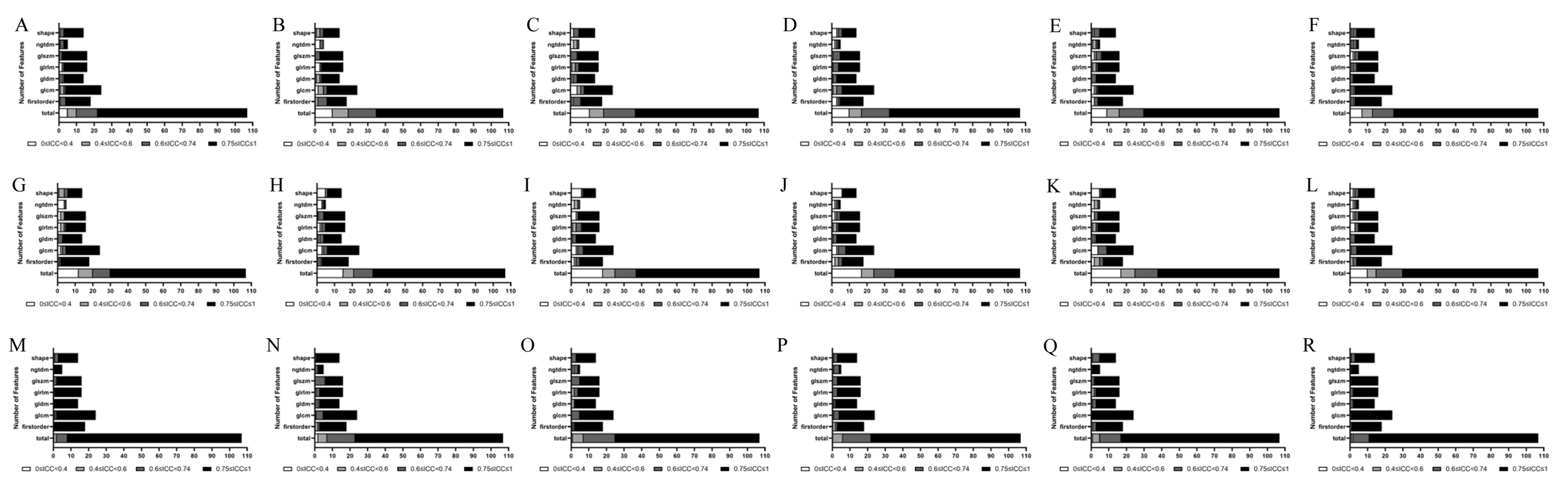

Supplement: Supplementary Figure 1 — The ICC values in each radiomic feature subgroup for CECT, MIP and MR delineations for tumor tissues. [file Image_1.jpg]

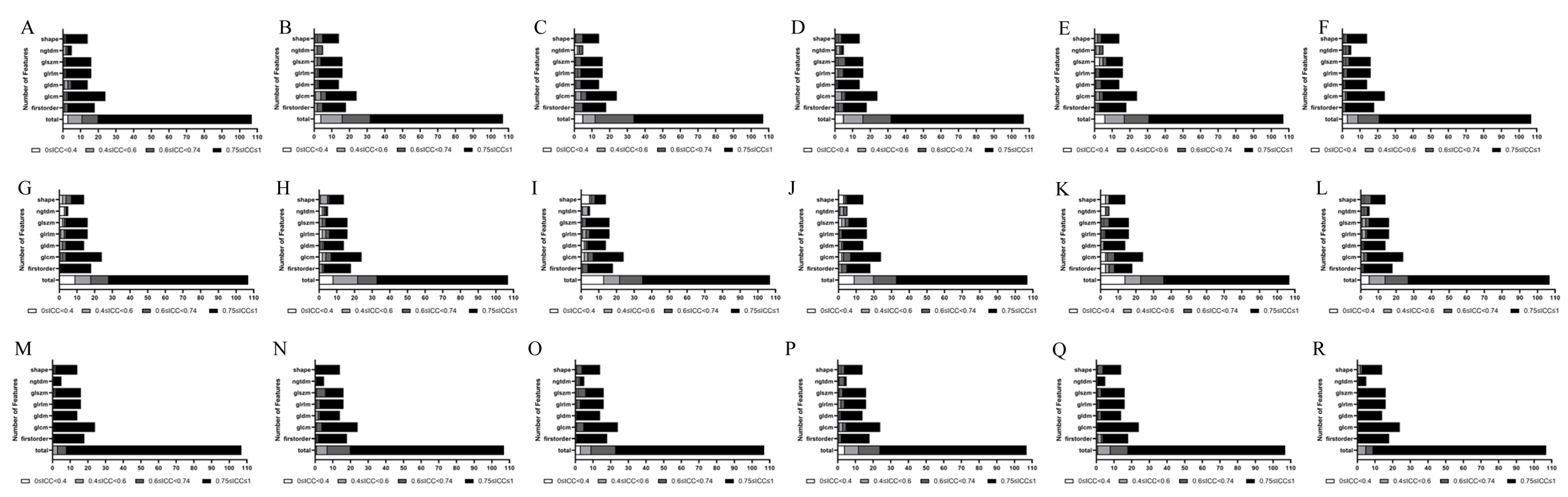

Supplement: Supplementary Figure 2 — The ICC values in each radiomic feature subgroup for CECT, MIP and MR delineations for peritumoral tissues. [file Image_2.jpg]
