# Supplementary material for: Reproducibility for Hepatocellular Carcinoma CT Radiomic Features: Influence of Delineation Variability Based on 3D-CT, 4D-CT and Multiple-Parameter MR Images
Source: Front Oncol. 2022 Apr 14;12:881931. doi: 10.3389/fonc.2022.881931 (PMC9047864; doi:10.3389/fonc.2022.881931)
Supplement: Supplementary Table 1 — The detailed QCD numbers of radiomic features. [file Table_1.docx]

| Observer | Fetaures  Subgroup | Variation Radiomics According to QCD (%)  Tumor tissues | | | | Variation Radiomics According to QCD (%)  Peritumoral tissues | | | |
| --- | --- | --- | --- | --- | --- | --- | --- | --- | --- |
|  |  | Very Small (QCD≤5%) | Small  (5%<QCD≤10%) | Intermediate  (10%<QCD≤20%) | Large  (QCD>20%) | Very Small (QCD≤5%) | Small  (5%<QCD≤10%) | Intermediate  (10%<QCD≤20%) | Large  (QCD>20%) |
| 3DCT1 | firstorder | 0.00%(0) | 1.87%(2) | 2.80%(3) | 12.15%(13) | 0.00%(0) | 0.93%(1) | 3.74%(4) | 12.15%(13) |
|  | glcm | 0.93%(1) | 2.80%(3) | 1.87%(2) | 16.82%(18) | 1.87%(2) | 2.80%(3) | 4.67%(5) | 13.08%(14) |
|  | gldm | 0.93%(1) | 0.93%(1) | 1.87%(2) | 9.35%(10) | 0.93%(1) | 0.93%(1) | 1.87%(2) | 9.35%(10) |
|  | glrlm | 1.87%(2) | 0.93%(1) | 1.87%(2) | 10.28%(11) | 1.87%(2) | 0.93%(1) | 2.80%(3) | 9.35%(10) |
|  | glszm | 0.00%(0) | 0.93%(1) | 2.80%(3) | 11.21%(12) | 0.00%(0) | 0.93%(1) | 2.80%(3) | 11.21%(12) |
|  | ngtdm | 0.00%(0) | 0.00%(0) | 0.93%(1) | 3.74%(4) | 0.00%(0) | 0.00%(0) | 1.87%(2) | 2.80%(3) |
|  | shape | 0.93%(1) | 1.87%(2) | 2.80%(3) | 7.48%(8) | 0.93%(1) | 0.93%(1) | 1.87%(2) | 9.35%(10) |
|  | total | 4.67%(5) | 9.35%(10) | 14.95%(16) | 71.03%(76) | 5.61%(6) | 7.48%(8) | 19.63%(21) | 67.29%(72) |
| 3DCT2 | firstorder | 0.00%(0) | 0.93%(1) | 3.74%(4) | 12.15%(13) | 0.00%(0) | 0.93%(1) | 2.80%(3) | 13.08%(14) |
|  | glcm | 1.87%(2) | 1.87%(2) | 1.87%(2) | 16.82%(18) | 0.93%(1) | 1.87%(2) | 3.74%(4) | 15.89%(17) |
|  | gldm | 0.93%(1) | 1.87%(2) | 1.87%(2) | 8.41%(9) | 0.93%(1) | 0.93%(1) | 2.80%(3) | 8.41%(9) |
|  | glrlm | 0.93%(1) | 0.00%(0) | 0.93%(1) | 13.08%(14) | 0.93%(1) | 0.00%(0) | 2.80%(3) | 11.21%(12) |
|  | glszm | 0.00%(0) | 0.93%(1) | 2.80%(3) | 11.21%(12) | 0.00%(0) | 0.93%(1) | 2.80%(3) | 11.21%(12) |
|  | ngtdm | 0.00%(0) | 0.00%(0) | 1.87%(2) | 2.80%(3) | 0.00%(0) | 0.00%(0) | 1.87%(2) | 2.80%(3) |
|  | shape | 0.93%(1) | 1.87%(2) | 1.87%(2) | 8.41%(9) | 0.93%(1) | 1.87%(2) | 1.87%(2) | 8.41%(9) |
|  | total | 4.67%(5) | 7.48%(8) | 14.95%(16) | 72.90%(78) | 3.74%(4) | 6.54%(7) | 18.69%(20) | 71.03%(76) |
| 3DCT3 | firstorder | 0.00%(0) | 0.93%(1) | 2.80%(3) | 13.08%(14) | 0.93%(1) | 0.93%(1) | 3.74%(4) | 11.21%(12) |
|  | glcm | 1.87%(2) | 1.87%(2) | 1.87%(2) | 16.82%(18) | 0.93%(1) | 2.80%(3) | 3.74%(4) | 14.95%(16) |
|  | gldm | 0.93%(1) | 0.93%(1) | 1.87%(2) | 9.35%(10) | 0.93%(1) | 0.93%(1) | 1.87%(2) | 9.35%(10) |
|  | glrlm | 0.93%(1) | 0.93%(1) | 0.93%(1) | 12.15%(13) | 0.93%(1) | 0.93%(1) | 0.93%(1) | 12.15%(13) |
|  | glszm | 0.00%(0) | 0.00%(0) | 1.87%(2) | 13.08%(14) | 0.00%(0) | 0.00%(0) | 2.80%(3) | 12.15%(13) |
|  | ngtdm | 0.00%(0) | 0.00%(0) | 0.93%(1) | 3.74%(4) | 0.00%(0) | 0.00%(0) | 1.87%(2) | 2.80%(3) |
|  | shape | 0.00%(0) | 0.93%(1) | 1.87%(2) | 10.28%(11) | 0.93%(1) | 0.93%(1) | 1.87%(2) | 9.35%(10) |
|  | total | 3.74%(4) | 5.61%(6) | 12.15%(13) | 78.50%(84) | 4.67%(5) | 6.54%(7) | 16.82%(18) | 71.96%(77) |
| 3DCT4 | firstorder | 0.00%(0) | 1.87%(2) | 2.80%(3) | 12.15%(13) | 0.93%(1) | 0.93%(1) | 3.74%(4) | 11.21%(12) |
|  | glcm | 1.87%(2) | 2.80%(3) | 2.80%(3) | 14.95%(16) | 1.87%(2) | 1.87%(2) | 4.67%(5) | 14.02%(15) |
|  | gldm | 0.93%(1) | 1.87%(2) | 1.87%(2) | 8.41%(9) | 0.93%(1) | 1.87%(2) | 2.80%(3) | 7.48%(8) |
|  | glrlm | 0.00%(0) | 0.93%(1) | 2.80%(3) | 11.21%(12) | 0.93%(1) | 0.93%(1) | 3.74%(4) | 9.35%(10) |
|  | glszm | 0.93%(1) | 0.00%(0) | 1.87%(2) | 12.15%(13) | 0.00%(0) | 0.93%(1) | 1.87%(2) | 12.15%(13) |
|  | ngtdm | 0.00%(0) | 0.93%(1) | 0.93%(1) | 2.80%(3) | 0.00%(0) | 0.00%(0) | 1.87%(2) | 2.80%(3) |
|  | shape | 0.93%(1) | 0.93%(1) | 2.80%(3) | 8.41%(9) | 0.93%(1) | 0.93%(1) | 2.80%(3) | 8.41%(9) |
|  | total | 4.67%(5) | 9.35%(10) | 15.89%(17) | 70.09%(75) | 5.61%(6) | 7.48%(8) | 21.50%(23) | 65.42%(70) |
| MIP1 | firstorder | 0.00%(0) | 0.93%(1) | 0.93%(1) | 14.95%(16) | 0.00%(0) | 0.93%(1) | 1.87%(2) | 14.02%(15) |
|  | glcm | 0.93%(1) | 1.87%(2) | 1.87%(2) | 17.76%(19) | 1.87%(2) | 1.87%(2) | 3.74%(4) | 14.95%(16) |
|  | gldm | 0.00%(0) | 0.93%(1) | 1.87%(2) | 10.28%(11) | 0.00%(0) | 0.93%(1) | 1.87%(2) | 10.28%(11) |
|  | glrlm | 0.93%(1) | 1.87%(2) | 1.87%(2) | 10.28%(11) | 0.93%(1) | 1.87%(2) | 1.87%(2) | 10.28%(11) |
|  | glszm | 0.93%(1) | 0.93%(1) | 2.80%(3) | 10.28%(11) | 0.93%(1) | 1.87%(2) | 2.80%(3) | 9.35%(10) |
|  | ngtdm | 0.00%(0) | 0.93%(1) | 0.93%(1) | 2.80%(3) | 0.00%(0) | 0.00%(0) | 0.93%(1) | 3.74%(4) |
|  | shape | 0.93%(1) | 1.87%(2) | 2.80%(3) | 7.48%(8) | 0.93%(1) | 0.93%(1) | 1.87%(2) | 9.35%(10) |
|  | total | 3.74%(4) | 9.35%(10) | 13.08%(14) | 73.83%(79) | 4.67%(5) | 8.41%(9) | 14.95%(16) | 71.96%(77) |
| MIP2 | firstorder | 0.93%(1) | 0.93%(1) | 0.93%(1) | 14.02%(15) | 0.93%(1) | 0.93%(1) | 2.80%(3) | 12.15%(13) |
|  | glcm | 1.87%(2) | 1.87%(2) | 1.87%(2) | 16.82%(18) | 1.87%(2) | 1.87%(2) | 3.74%(4) | 14.95%(16) |
|  | gldm | 0.00%(0) | 0.93%(1) | 1.87%(2) | 10.28%(11) | 0.00%(0) | 0.93%(1) | 1.87%(2) | 10.28%(11) |
|  | glrlm | 0.00%(0) | 0.93%(1) | 1.87%(2) | 12.15%(13) | 0.93%(1) | 0.93%(1) | 2.80%(3) | 10.28%(11) |
|  | glszm | 0.93%(1) | 0.93%(1) | 1.87%(2) | 11.21%(12) | 0.93%(1) | 0.00%(0) | 1.87%(2) | 12.15%(13) |
|  | ngtdm | 0.00%(0) | 0.00%(0) | 0.93%(1) | 3.74%(4) | 0.00%(0) | 0.00%(0) | 1.87%(2) | 2.80%(3) |
|  | shape | 0.93%(1) | 1.87%(2) | 2.80%(3) | 7.48%(8) | 0.00%(0) | 1.87%(2) | 2.80%(3) | 8.41%(9) |
|  | total | 4.67%(5) | 7.48%(8) | 12.15%(13) | 75.70%(81) | 4.67%(5) | 6.54%(7) | 17.76%(19) | 71.03%(76) |
| MIP3 | firstorder | 0.00%(0) | 0.93%(1) | 1.87%(2) | 14.02%(15) | 0.93%(1) | 0.93%(1) | 2.80%(3) | 12.15%(13) |
|  | glcm | 0.93%(1) | 1.87%(2) | 1.87%(2) | 17.76%(19) | 0.93%(1) | 1.87%(2) | 3.74%(4) | 15.89%(17) |
|  | gldm | 0.00%(0) | 0.00%(0) | 0.93%(1) | 12.15%(13) | 0.00%(0) | 0.93%(1) | 1.87%(2) | 10.28%(11) |
|  | glrlm | 0.93%(1) | 1.87%(2) | 0.93%(1) | 11.21%(12) | 0.93%(1) | 1.87%(2) | 0.93%(1) | 11.21%(12) |
|  | glszm | 0.93%(1) | 0.93%(1) | 0.93%(1) | 12.15%(13) | 0.93%(1) | 0.93%(1) | 1.87%(2) | 11.21%(12) |
|  | ngtdm | 0.00%(0) | 0.00%(0) | 1.87%(2) | 2.80%(3) | 0.00%(0) | 0.00%(0) | 1.87%(2) | 2.80%(3) |
|  | shape | 0.93%(1) | 0.93%(1) | 1.87%(2) | 9.35%(10) | 0.93%(1) | 0.00%(0) | 2.80%(3) | 9.35%(10) |
|  | total | 3.74%(4) | 6.54%(7) | 10.28%(11) | 79.44%(85) | 4.67%(5) | 6.54%(7) | 15.89%(17) | 72.90%(78) |
| MIP4 | firstorder | 0.93%(1) | 0.93%(1) | 1.87%(2) | 13.08%(14) | 0.93%(1) | 0.93%(1) | 3.74%(4) | 11.21%(12) |
|  | glcm | 1.87%(2) | 1.87%(2) | 2.80%(3) | 15.89%(17) | 1.87%(2) | 1.87%(2) | 4.67%(5) | 14.02%(15) |
|  | gldm | 0.00%(0) | 0.00%(0) | 1.87%(2) | 11.21%(12) | 0.00%(0) | 0.00%(0) | 2.80%(3) | 10.28%(11) |
|  | glrlm | 0.93%(1) | 0.93%(1) | 1.87%(2) | 11.21%(12) | 0.93%(1) | 0.93%(1) | 1.87%(2) | 11.21%(12) |
|  | glszm | 0.93%(1) | 1.87%(2) | 1.87%(2) | 10.28%(11) | 0.00%(0) | 0.93%(1) | 2.80%(3) | 11.21%(12) |
|  | ngtdm | 0.00%(0) | 0.00%(0) | 1.87%(2) | 2.80%(3) | 0.00%(0) | 0.00%(0) | 1.87%(2) | 2.80%(3) |
|  | shape | 0.93%(1) | 0.93%(1) | 1.87%(2) | 9.35%(10) | 0.93%(1) | 0.93%(1) | 3.74%(4) | 7.48%(8) |
|  | total | 5.61%(6) | 6.54%(7) | 14.02%(15) | 73.83%(79) | 4.67%(5) | 5.61%(6) | 21.50%(23) | 68.22%(73) |
| MR1 | firstorder | 0.93%(1) | 0.93%(1) | 2.80%(3) | 12.15%(13) | 0.93%(1) | 0.93%(1) | 2.80%(3) | 12.15%(13) |
|  | glcm | 1.87%(2) | 1.87%(2) | 3.74%(4) | 14.95%(16) | 1.87%(2) | 1.87%(2) | 4.67%(5) | 14.02%(15) |
|  | gldm | 0.00%(0) | 0.00%(0) | 2.80%(3) | 10.28%(11) | 0.00%(0) | 0.00%(0) | 2.80%(3) | 10.28%(11) |
|  | glrlm | 0.93%(1) | 1.87%(2) | 2.80%(3) | 9.35%(10) | 0.93%(1) | 1.87%(2) | 2.80%(3) | 9.35%(10) |
|  | glszm | 0.93%(1) | 1.87%(2) | 1.87%(2) | 10.28%(11) | 0.93%(1) | 0.93%(1) | 2.80%(3) | 10.28%(11) |
|  | ngtdm | 0.00%(0) | 0.93%(1) | 0.93%(1) | 2.80%(3) | 0.00%(0) | 0.93%(1) | 1.87%(2) | 1.87%(2) |
|  | shape | 0.93%(1) | 0.93%(1) | 2.80%(3) | 8.41%(9) | 0.00%(0) | 0.93%(1) | 2.80%(3) | 9.35%(10) |
|  | total | 5.61%(6) | 8.41%(9) | 17.76%(19) | 68.22%(73) | 4.67%(5) | 7.48%(8) | 20.56%(22) | 67.29%(72) |
| MR2 | firstorder | 0.93%(1) | 0.93%(1) | 2.80%(3) | 12.15%(13) | 0.93%(1) | 1.87%(2) | 2.80%(3) | 11.21%(12) |
|  | glcm | 0.93%(1) | 1.87%(2) | 3.74%(4) | 15.89%(17) | 1.87%(2) | 2.80%(3) | 4.67%(5) | 13.08%(14) |
|  | gldm | 0.00%(0) | 0.00%(0) | 1.87%(2) | 11.21%(12) | 0.00%(0) | 0.00%(0) | 1.87%(2) | 11.21%(12) |
|  | glrlm | 0.93%(1) | 1.87%(2) | 2.80%(3) | 9.35%(10) | 0.93%(1) | 1.87%(2) | 1.87%(2) | 10.28%(11) |
|  | glszm | 0.93%(1) | 0.93%(1) | 1.87%(2) | 11.21%(12) | 0.93%(1) | 1.87%(2) | 2.80%(3) | 9.35%(10) |
|  | ngtdm | 0.00%(0) | 0.00%(0) | 1.87%(2) | 2.80%(3) | 0.00%(0) | 0.00%(0) | 1.87%(2) | 2.80%(3) |
|  | shape | 0.93%(1) | 0.93%(1) | 2.80%(3) | 8.41%(9) | 0.93%(1) | 0.93%(1) | 1.87%(2) | 9.35%(10) |
|  | total | 4.67%(5) | 6.54%(7) | 17.76%(19) | 71.03%(76) | 5.61%(6) | 9.35%(10) | 17.76%(19) | 67.29%(72) |
| MR3 | firstorder | 0.00%(0) | 1.87%(2) | 2.80%(3) | 12.15%(13) | 0.93%(1) | 1.87%(2) | 3.74%(4) | 10.28%(11) |
|  | glcm | 0.93%(1) | 1.87%(2) | 3.74%(4) | 15.89%(17) | 1.87%(2) | 1.87%(2) | 3.74%(4) | 14.95%(16) |
|  | gldm | 0.00%(0) | 0.00%(0) | 1.87%(2) | 11.21%(12) | 0.93%(1) | 0.00%(0) | 2.80%(3) | 9.35%(10) |
|  | glrlm | 0.93%(1) | 1.87%(2) | 2.80%(3) | 9.35%(10) | 0.00%(0) | 0.93%(1) | 2.80%(3) | 11.21%(12) |
|  | glszm | 0.93%(1) | 0.93%(1) | 1.87%(2) | 11.21%(12) | 0.93%(1) | 0.93%(1) | 1.87%(2) | 11.21%(12) |
|  | ngtdm | 0.00%(0) | 0.00%(0) | 1.87%(2) | 2.80%(3) | 0.00%(0) | 0.00%(0) | 2.80%(3) | 1.87%(2) |
|  | shape | 0.93%(1) | 0.93%(1) | 2.80%(3) | 8.41%(9) | 0.93%(1) | 0.93%(1) | 2.80%(3) | 8.41%(9) |
|  | total | 3.74%(4) | 7.48%(8) | 17.76%(19) | 71.03%(76) | 5.61%(6) | 6.54%(7) | 20.56%(22) | 67.29%(72) |
| MR4 | firstorder | 0.00%(0) | 1.87%(2) | 2.80%(3) | 12.15%(13) | 0.93%(1) | 0.93%(1) | 2.80%(3) | 12.15%(13) |
|  | glcm | 1.87%(2) | 1.87%(2) | 3.74%(4) | 14.95%(16) | 0.93%(1) | 1.87%(2) | 3.74%(4) | 15.89%(17) |
|  | gldm | 0.00%(0) | 0.00%(0) | 1.87%(2) | 11.21%(12) | 0.93%(1) | 0.93%(1) | 1.87%(2) | 9.35%(10) |
|  | glrlm | 0.93%(1) | 0.93%(1) | 3.74%(4) | 9.35%(10) | 0.93%(1) | 0.00%(0) | 2.80%(3) | 11.21%(12) |
|  | glszm | 0.93%(1) | 0.93%(1) | 1.87%(2) | 11.21%(12) | 0.93%(1) | 1.87%(2) | 1.87%(2) | 10.28%(11) |
|  | ngtdm | 0.00%(0) | 0.00%(0) | 2.80%(3) | 1.87%(2) | 0.00%(0) | 0.93%(1) | 1.87%(2) | 1.87%(2) |
|  | shape | 0.93%(1) | 0.93%(1) | 2.80%(3) | 8.41%(9) | 0.00%(0) | 0.93%(1) | 2.80%(3) | 9.35%(10) |
|  | total | 4.67%(5) | 6.54%(7) | 19.63%(21) | 69.16%(74) | 4.67%(5) | 7.48%(8) | 17.76%(19) | 70.09%(75) |
