# Supplementary material for: Reproducibility for Hepatocellular Carcinoma CT Radiomic Features: Influence of Delineation Variability Based on 3D-CT, 4D-CT and Multiple-Parameter MR Images
Source: Front Oncol. 2022 Apr 14;12:881931. doi: 10.3389/fonc.2022.881931 (PMC9047864; doi:10.3389/fonc.2022.881931)
Supplement: Supplementary Table 2 — The specific radiomic feature with small variation and excellent reproducibility. [file Table_2.docx]

|  | Tumor | Peritumoral |
| --- | --- | --- |
| ICC>0.75 | firstorder-10Percentile; firstorder-90Percentile; firstorder-Energy; firstorder-Entropy; firstorder-Maximum; firstorder-Mean; firstorder-MeanAbsoluteDeviation; firstorder-Median; firstorder-RobustMeanAbsoluteDeviation; firstorder-RootMeanSquared; firstorder-Variance; glcm-Contrast; glcm-Correlation; glcm-DifferenceEntropy; glcm-Idm; glcm-Imc2; glcm-JointAverage; glcm-MaximumProbability; glcm-MCC; glcm-SumAverage; glcm-SumEntropy; gldm-DependenceEntropy; gldm-DependenceNonUniformity; gldm-DependenceNonUniformityNormalized; gldm-DependenceVariance; gldm-GrayLevelVariance; gldm-LargeDependenceEmphasis; gldm-LargeDependenceLowGrayLevelEmphasis; gldm-SmallDependenceEmphasis; gldm-SmallDependenceHighGrayLevelEmphasis; glrlm-GrayLevelNonUniformityNormalized; glrlm-LongRunEmphasis; glrlm-RunLengthNonUniformityNormalized; glrlm-RunPercentage; glrlm-ShortRunEmphasis; glszm-HighGrayLevelZoneEmphasis; glszm-LargeAreaEmphasis; glszm-LargeAreaLowGrayLevelEmphasis; glszm-SizeZoneNonUniformity; glszm-ZonePercentage; ngtdm-Contrast; shape-MajorAxisLength; shape-Maximum2DDiameterColumn; shape-Maximum3DDiameter; shape-MeshVolume; shape-SurfaceArea; shape-SurfaceVolumeRatio | firstorder-InterquartileRange; firstorder-Median; firstorder-Minimum; firstorder-Range; firstorder-RobustMeanAbsoluteDeviation; firstorder-RootMeanSquared; firstorder-TotalEnergy; firstorder-Uniformity; glcm-ClusterProminence; glcm-ClusterShade; glcm-ClusterTendency; glcm-Correlation; glcm-Id; glcm-Idmn; glcm-Imc1; glcm-JointEnergy; glcm-JointEntropy; glcm-MCC; glcm-SumAverage; gldm-DependenceEntropy; gldm-DependenceNonUniformity; gldm-DependenceNonUniformityNormalized; gldm-DependenceVariance; gldm-GrayLevelVariance; gldm-LargeDependenceHighGrayLevelEmphasis; gldm-LargeDependenceLowGrayLevelEmphasis; glrlm-GrayLevelVariance; glrlm-LongRunEmphasis; glrlm-LongRunHighGrayLevelEmphasis; glrlm-LowGrayLevelRunEmphasis; glrlm-ShortRunEmphasis; glrlm-ShortRunLowGrayLevelEmphasis; glszm-LargeAreaHighGrayLevelEmphasis; glszm-SmallAreaLowGrayLevelEmphasis; glszm-ZoneEntropy; ngtdm-Contrast; shape-Flatness; shape-LeastAxisLength; shape-Maximum2DDiameterColumn; shape-MinorAxisLength |
| QCD<10% | firstorder-Entropy; glcm-Id; glcm-Idm; glcm-Idmn; glcm-Idn; glcm-JointEntropy; gldm-DependenceEntropy; gldm-DependenceNonUniformityNormalized; glrlm-RunEntropy; glrlm-ShortRunEmphasis | firstorder-Entropy; firstorder-Median; glcm-Id; glcm-Idm; glcm-Idmn; glcm-Idn; glcm-SumEntropy; gldm-DependenceEntropy; glrlm-ShortRunEmphasis; glszm-SmallAreaEmphasis; glszm-ZoneEntropy |
| ICC>0.75 in tumor and peritumoral | firstorder-Median; firstorder-RobustMeanAbsoluteDeviation; firstorder-RootMeanSquared; glcm-Correlation; glcm-MCC; glcm-SumAverage; gldm-DependenceEntropy; gldm-DependenceNonUniformity; gldm-DependenceNonUniformityNormalized; gldm-DependenceVariance; gldm-GrayLevelVariance; gldm-LargeDependenceLowGrayLevelEmphasis; glrlm-ShortRunEmphasis; ngtdm-Contrast; shape-Maximum2DDiameterColumn | |
| QCD<10% in tumor and peritumoral | firstorder-Entropy; glcm-Id; glcm-Idm; glcm-Idmn; glcm-Idn; gldm-DependenceEntropy; glrlm-ShortRunEmphasis | |
| ICC>0.75& QCD<10% in tumor and peritumoral | gldm-DependenceEntropy; glrlm-ShortRunEmphasis | |
